# Exposure of *Trypanosoma brucei* to an *N*-acetylglucosamine-Binding Lectin Induces VSG Switching and Glycosylation Defects Resulting in Reduced Infectivity

**DOI:** 10.1371/journal.pntd.0003612

**Published:** 2015-03-06

**Authors:** Víctor M. Castillo-Acosta, Luis M. Ruiz-Pérez, Els J. M. Van Damme, Jan Balzarini, Dolores González-Pacanowska

**Affiliations:** 1 Instituto de Parasitología y Biomedicina “López-Neyra”, Consejo Superior de Investigaciones Científicas, Parque Tecnológico de Ciencias de la Salud, Granada, Spain; 2 Laboratory of Biochemistry and Glycobiology, Department of Molecular Biotechnology, Ghent University, Ghent, Belgium; 3 Rega Institute for Medical Research, KU Leuven, Leuven, Belgium; New York University School of Medicine, UNITED STATES

## Abstract

*Trypanosoma brucei* variant surface glycoproteins (VSG) are glycosylated by both paucimannose and oligomannose structures which are involved in the formation of a protective barrier against the immune system. Here, we report that the stinging nettle lectin (UDA), with predominant *N*-acetylglucosamine-binding specificity, interacts with glycosylated VSGs and kills parasites by provoking defects in endocytosis together with impaired cytokinesis. Prolonged exposure to UDA induced parasite resistance based on a diminished capacity to bind the lectin due to an enrichment of biantennary paucimannose and a reduction of triantennary oligomannose structures. Two molecular mechanisms involved in resistance were identified: VSG switching and modifications in *N*-glycan composition. Glycosylation defects were correlated with the down-regulation of the *TbSTT3A* and/or *TbSTT3B* genes (coding for oligosaccharyltransferases A and B, respectively) responsible for glycan specificity. Furthermore, UDA-resistant trypanosomes exhibited severely impaired infectivity indicating that the resistant phenotype entails a substantial fitness cost. The results obtained further support the modification of surface glycan composition resulting from down-regulation of the genes coding for oligosaccharyltransferases as a general resistance mechanism in response to prolonged exposure to carbohydrate-binding agents.

## Author Summary

*Trypanosoma brucei*, the causative agent of African trypanosomiasis, is covered by glycosylphosphatidylinositol-anchored glycoproteins, which shield parasites from effectors of the host immune system. The most abundant protein is the variant surface glycoprotein (VSG), which plays an essential role in antigenic variation and the ability of the parasite to evade the immune system. VSGs are *N*-glycosylated in a site-specific manner by different oligosaccharyltransferases giving rise to complex and mannose-rich *N*-glycans. In this study, we report that the carbohydrate binding agent, stinging nettle agglutinin (UDA, *Urtica dioica*), exhibits trypanocidal activity, impairs endocytosis, and cytokinesis and induces parasite lysis. We have investigated the mechanisms conferring resistance in order to understand UDA mode of action and found that resistant strains present changes in the expression of oligosaccharyltransferases, and modifications in the nature of the predominant VSG, therefore resulting in important changes in *N*-glycan composition. The modification in surface glycans gives rise to reduced infectivity *in vivo* further underscoring the role of glycosylation in parasite virulence. Our findings show that carbohydrate binding agents that bind effectively to surface glycoproteins can provide a novel avenue for design of drugs to combat African trypanosomiasis.

## Introduction

*Trypanosoma brucei* spp are protozoan parasites that cause the neglected disease known as African trypanosomiasis or sleeping sickness in human and nagana in animals, which exhibit a fatal impact on health and economy of the affected countries. So far, the treatments are insufficient and unsatisfactory; therefore discovery of new approaches to design novel drugs is a critical challenge to combat these diseases.

Bloodstream forms of the parasites living in the mammalian host rely on antigenic variation to evade the immune system of the host. The parasites are mainly covered by a single type of variant surface glycoprotein (VSG) at a given moment during infection. Thus, trypanosomes build an effective barrier that protects other invariant proteins of their surface from effectors of the host immune system. A major contribution to the formation of this protective barrier are the *N*-glycans of the VSGs [1], which contain specific triantennary oligomannose or biantennary paucimannose structures [2–4]. The *N*-glycosylation of the VSG in *T*. *brucei* is made in a site-specific manner by the action of distinct oligosaccharyltransferase (OST) activities coded by three *STT3A*, *STT3B* and *STT3C* genes [5–7]. For instance, in VSG221, TbSTT3A transfers principally the Man_5_GlcNAc_2_-PP-Dol to asparagines (Asn263), flanked by an acidic sequence, whereas TbSTT3B transfers primarily the Man_9_GlcNAc_2_-PP-Dol to any remaining asparagine (Asn428). The Man_5_GlcNAc_2_-PP-Dol structures are responsible of all paucimannose and complex *N*-glycans, while the Man_9_GlcNAc_2_-PP-Dol structures are responsible of all oligomannose-rich *N*-glycans [7]. *N*-acetylglucosamine (GlcNAc) is the common core of all *N*-glycans (paucimannose and oligomannose structures) and is also present in hybrid and complex glycans.

*T*. *brucei* exhibits a mechanism known as VSG switching by which parasites change the surface coat expressing a new VSG from the massive battery of VSG genes and consequently evade the immune system. This process can occur by gene conversion (DNA rearrangement) which involves recombination of a silent *VSG* gene into the active expression site (ES), or *in situ* switch (transcriptional control) where the exclusive actively transcribed ES is silenced with the subsequent activation of a silent ES [8]. Antibodies can recognize efficiently VSGs but *T*. *brucei* can overcome agglutination through efficient endocytosis of antibody-VSG complexes [9–11], avoiding a rapid antibody-dependent complement-directed lysis [12,13]. Subsequently, in sorting endosomes, the VSG is detached from antibodies and translocated to surface parasites by the recycling endosomes [14–17].

Recently, a series of carbohydrate-binding agents (CBAs) have been reported as strong antiviral agents with a dual mechanism of action againts human immunodeficiency virus (HIV) and human hepatitis C virus (HCV) [18]. CBAs directly blocks virus entry to the target cells by binding to glycans of the viral envelope, and indirectly allowing the immune system to recognize previously hidden immunogenic epitopes by progressive glycan deletions in the envelope glycoprotein. As mentioned above, *T*. *brucei* is mainly covered by VSGs which harbour *N*-glycans susceptible to bind CBAs.

We have previously reported that a series of CBAs exhibit strong trypanocidal activity against the clinically relevant bloodstream form, with 50% effective concentration (EC_50s_) values in the nanomolar range [19]. We now show that the stinging nettle lectin from *Urtica dioica* (UDA) binds to the surface glycans in an irreversible fashion, inhibiting profoundly endocytosis, affecting cell cycle progression and killing parasites. Furthermore, prolonged exposure of parasites to UDA *in vitro* gave rise to resistance by different mechanisms including VSG switching and changes in the *N*-glycosylation profile. Resistance was associated with modifications in the expression levels of the *STT3* genes, leading to a strong fitness cost in mouse models behaving in some cases as non-infectious. The present evidence further revealed that interfering with the pattern and accessibility of *N*-glycans of surface glycoproteins may provide novel avenues for therapeutic intervention.

## Materials and Methods

### Ethics statement

The animal research described in this manuscript complied with Spanish (Ley 32/2007) and European Union Legislation (2010/ 63/UE). The protocols used were approved by the Animal Care Committee of the Instituto de Parasitología y Biomedicina “López-Neyra”, CSIC (protocol 2738/13.A.2).

### Trypanosome cultures

*Trypanosoma brucei* single-marker bloodstream form (BSF) was used in this study, which expresses the VSG221 also called MITat 1.2 [20]. Trypanosomes were cultured at 37°C and 5% CO_2_ in HMI-9 with 10% (v/v) fetal bovine serum.

### Carbohydrate-binding agents

The peptidic CBAs used in this study were the stinging nettle agglutinin (UDA, *Urtica dioica*) [21], *Hippeastrum* hybrid agglutinin (HHA) [22], broad-leaved helleborine agglutinin (EHA, *Epipactis helleborine*) [23], snowdrop agglutinin (GNA, *Galanthus nivalis*) [24] and daffodil agglutinin (NPA, *Narcissus pseudonarcissus*) [25]. Concanavalin A (ConA) was obtained from Sigma and tomato lectin (TL) from Vector Laboratories, Inc. UDA was first purified by Peumans et al. [21] from stinging nettle rhizomes; the protocol was later refined as described in Van Damme *et al*. [26]. The purity of the protein was checked by SDS-PAGE and also by mass spectrometry. The same purification protocol was also used to obtain lectin preparations used for crystallization and analysis of the three-dimensional structure of UDA [27], indicating the high purity of the lectin preparation.

### Generation of UDA-resistant cell lines

UDA is a lectin isolated from *Urtica dioica* with predominant *N*-acetylglucosamine (GlcNAc)-specificity, and was used to generate resistant cell lines of the *T*. *brucei* bloodstream form at escalating concentrations. Two lines of parasite mutants (UDAa and UDAb) were obtained at the same concentrations, starting at the EC_50_ value (2.0 μg/mL, 0.225 μM) and followed by consecutive selection rounds. Parasites were exposed to the next higher concentration (3.5, 5, 10 and 15 μg/mL) when the generation time had equaled that of the parental cell line (6–8 hours), which took around 20–40 days. To evaluate the stability of the drug resistance, mutant parasites of the last two strains were grown during 1, 2 or 3 months in the absence of UDA.

### Immunofluorescence analysis of VSG expression

UDA-resistant cells were subjected to immunofluorescence studies using an anti-TbVSG221 polyclonal antibody to analyze the expression of VSG221. Thus, trypanosomes were fixed on poly-L-lysine-coated slides with 4% *p-*formaldehyde, washed twice (PBS and 0.2% Tween 20) and blocked during 30 min with Blocking Reagent 1% (Roche). Next, samples were incubated with anti-TbVSG221 antibody for 1 h, washed, and probed with FITC-conjugated anti-rabbit secondary antibody for 1 h. After washing, slides were dehydrated in methanol for 1 min, stained and mounted with Vectashield-DAPI (Vector Laboratories, Inc.). The microscopy and digital image acquisition were carried out with a Zeiss Axiophot microscope (Carl Zeiss, Inc.)

### RNA extraction and cDNA synthesis

Total RNA of parental and UDA-resistant cell lines was extracted using TRIzol reagent (Invitrogen), and subsequently treated with DNase by the RNeasy Micro kit (Qiagen) to remove remaining genomic DNA. cDNA was obtained by reverse transcription using iScript cDNA synthesis kit. All procedures were performed according to the manufacturer’s instructions.

### Real time quantitative PCR (RT-qPCR)

mRNA expression levels of *TbSTT3A*, *TbSTT3B* and *TbSTT3C* genes were evaluated by RT-qPCR using SsoFast^™^ EvaGreen Supermix (Bio-Rad) in an iCycler IQ real-time PCR detection system (Bio-Rad), according to the manufacturer's recommendations. Relative expression of these genes as well as *myosin 1B* gene (Tb11.01.7990) was calculated relative to the mRNA expression levels of the *PI3K-like* gene (Tb927.2.2260) as previously described [19]. Three independent experiments were performed and the sample triplicates were used in all RT-qPCR assays.

### Sequencing of VSG221 and the VSG active expression site

Sequences of the VSGs active expression site of the UDAa strains were analyzed to establish the mechanism involved in the switching events. Thus, a specific downstream region of the VSG promoters was amplified (344 bp) from UDAa-resistant and parental strains cDNA. Primers used were: 5’-GGC GGA CGT CTC GAA CCG ATC GTG AG-3’ (forward) and 5’-TAA CCC TCA CAA TCT CCG ATC ATG C-3’ (reverse) [28].

VSG221 sequences of UDA10b and UDA15b strains were analyzed to evaluate potential mutations in asparagine residues as responsible for the *N*-glycosylation defects. For this purpose, PCR was performed from cDNA using a pair of specific primers to amplify the open reading frame of the VSG221. In both cases, the amplified fragments were cloned in the pGEM-T vector (Promega) and subsequently sequenced.

### Small-scale sVSG (soluble-form VSG) isolation and endoglycosidase digestion

The sVSG isolation of UDA-resistant strains was performed following the protocol described by Cross et al [29,30] with slight modifications. Thus, pellets from 2 x 10^8^ cells were lysed in 300 μL of hypotonic lysis buffer (10 mM sodium phosphate buffer, pH 8.0) supplemented with protease inhibitor cocktail (Roche) for 5 min at 37°C. After centrifugation at 14,000 x *g* for 5 min, the supernatant was loaded onto 0.2 mL of DE52 (Whatman), and VSG was eluted with 0.8 mL of hypotonic lysis buffer. Finally, VSG was resuspended in water after diafiltering on a Nanosep 10K Omega (Pall Corporation).

Endoglycosidase digestion was performed on sVSGs of UDA-resistant strains. For each enzyme digestion, 1 μg of sVSG was denatured in 10 μL of 0.5% SDS and 0.1 M DTT at 100°C for 10 min, and treated overnight with 500 units of Endo H or PNGase F (New England Biolabs) at 37°C, in the corresponding buffer supplied by the manufacturer.

### Assessment of endocytosis by flow cytometry and immunofluorescence microscopy

UDA was conjugated to the FITC dye using the FluoReporter FITC Protein Labeling Kit (Molecular Probes, Inc.), according to the manufacturer’s instructions. Live parental *T*. *brucei* cells (1.5 x 10^6^ parasites) were washed once with Voorheis PBS (PBS containing 10 mM glucose and 79 mM sucrose), resuspended in 1 mL of serum-free HMI-9 medium containing 1% BSA and preincubated at 37°C for 20 min. Next, samples were incubated at increasing times with FITC-conjugated UDA (1 mg/mL), washed twice with cold PBS and finally resuspended in PBS. FACS analysis was carried out using a Becton Dickinson FACSCalibur and BD CellQuest Pro version 4.0.2 software. In the case of microscopy analysis, cells were fixed with 2% *p*-formaldehyde at 4°C for 1 h, washed, and adhered on poly-L-lysine coated slides. Finally, slides were dehydrated in methanol for 1 min and stained with Vectashield-DAPI (Vector Laboratories, Inc.). Vertical stacks of 15–25 slices (0.15 μm steps) were captured using an Olympus microscope and Cell R IX81 software. Deconvolution and pseudo-colouring of images was performed using Huygens Essential software (version 3.3; Scientific Volume Imaging) and Image J software (version 1.37; National Institutes of Health), respectively.

In addition, fixed cells were labeled with UDA-FITC conjugates as described above for immunofluorescence analysis. Microscopy and digital image acquisition were carried out with a Zeiss Axiophot microscope (Carl Zeiss, Inc.)

FITC-transferrin conjugates (50 μg/mL) and ConA-AlexaFluor 594 conjugates (100 μg/mL) (Molecular Probes, Invitrogen detection Technologies) were used to establish the endocytosis dynamics in UDA-resistant strains. Alexa Fluor 488-labeled dextran 10,000 (2.5 mg/mL) (Molecular Probes) was used to evaluate fluid-phase endocytosis. Uptake assays were performed as indicated for UDA-FITC labeling, with the difference that the incubation with dextran was done in a final volume of 20 μL. Fluorescence was measured by flow cytometry using a Becton Dickinson FACSCalibur and BD CellQuest Pro version 4.0.2 software.

### Lectin blotting

The glycosylation profile of UDA15a and UDA15b cell lines was studied by lectin blotting using ConA and TL. Both, sVSG (2 μg) and cell pellets (1 x 10^6^ cell equivalents/sample) coming from hypotonic lysis were denatured in SDS-sample buffer containing 8 M urea and 50 mM DTT, subjected to electrophoresis on a NuPAGE Bis-Tris 4–12% gradient gel (Invitrogen) in MOPS buffer and transferred to a nitrocellulose membrane. Proteins were stained with Ponceau S (Sigma) as loading control and blocked with 3% BSA in PBS. Membranes were probed with biotinylated TL (0.33 μg/mL, Vector Laboratories, Inc.) in a solution containing 50 mM Tris-HCl pH 7.4, 0.5 M NaCl, 0.05% IGEPAL and 0.25% BSA, or biotinylated ConA (0.05 μg/mL, Sigma) in PBS containing 1 mM MgCl_2_, 1 mM CaCl_2_, 1 mM MnCl_2_, 0.05% IGEPAL and 0.25% BSA. Specific inhibitors of lectin binding such as chitin hydrolysate (1:10 dilution, Vector Laboratories, Inc.) for TL and methyl α-D- mannopyranoside (0.5 M, Sigma) for ConA were also used as carbohydrate-specific binding controls. Finally, glycoproteins were detected with Extravidin-peroxidase conjugated (Sigma) by chemiluminescent detection ECL Western Blotting Detection Reagents (GE Healthcare).

### *In vivo* studies

Three female Balb/C mice per group (6–8 weeks old) were infected via intraperitoneal *route* with 600 monomorphic *T*. *brucei* parasites from UDA15a-resistant, UDA15b-resistant and parental cell lines. The infection course was monitored by parasitaemia counted in a haematocytometer under a microscope after tail blood extraction.

### Statistical analysis

We expressed the results as the mean ± SD for each group, and comparisons between groups were performed using Student’s *t*-tests using a commercially available, computer-based statistical package (GraphPad Software Inc.) for all calculations. A *p* value ≤0.05 was considered statistically significant.

## Results

### UDA binds irreversibly to the surface coat of trypanosomes inducing cell cycle defects and cell death

**Fig 1:**
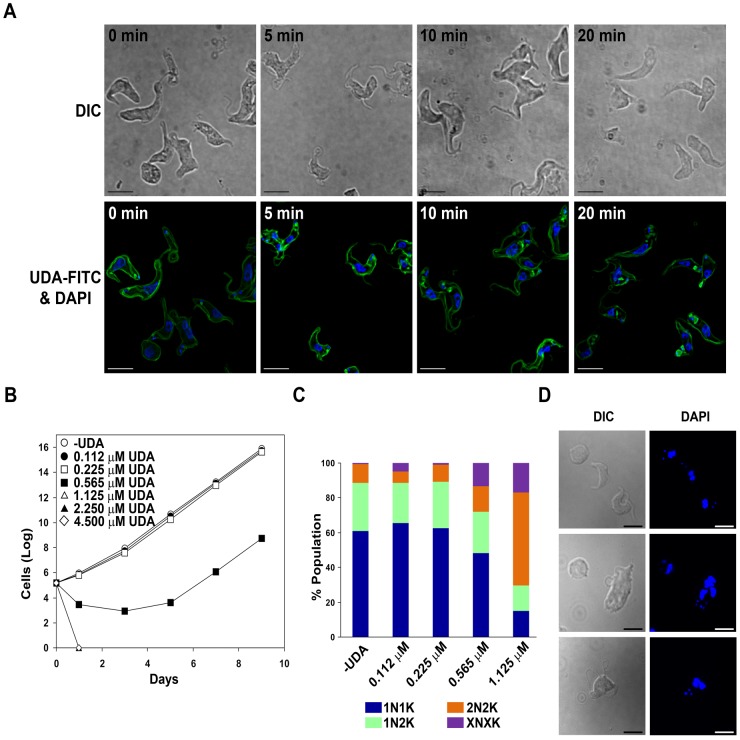
UDA binding to the surface coat of *T*. *brucei*. A) 3D microscopy images of *T*. *brucei* BSF live cells labeled with UDA-FITC conjugates for different time periods. B) Accumulated growth of *T*. *brucei* BSF parasites after exposure to different concentrations of UDA for 24 h. C) Nuclei (N) and kinetoplasts (K) were stained with DAPI and categorized according to their number in 1N1K, 1N2K, 2N2K in addition to a population with an aberrant number of nuclei and kinetoplast denoted by XNXK. D) Microscopy images showing DAPI staining and cell morphology after UDA exposure for 10 h. Bars, 10 μm.

We have recently reported a new series of CBAs exhibiting trypanocidal activity in the bloodstream forms [19] in contrast with the established idea that *T*. *brucei* does not bind efficiently lectins or that they are degraded in the lysosome after glycoprotein-lectin complex internalization [31]. Thus, *Urtica dioica* agglutinin (UDA), one of the smallest monomeric plant lectins isolated from stinging nettle root (≈ 8.7 kDa), shows a predominant specificity for *N*-acetylglucosamine (GlcNAc) with pronounced antiviral properties [23]. In the case of bloodstream forms of *T*. *brucei*, we reported that UDA exhibits a significant trypanocidal activity (EC_50_ of 0.225 μM) [19]. To determine the physiological target of this lectin, we first used a UDA-FITC conjugate to label bloodstream live parasites. The cellular localization was monitored for different time periods by fluorescence 3D microscopy. UDA was found predominantly on the surface coat, and also appeared in the flagellar pocket yet minor amounts seemed to be internalized via the endocytic pathway suggesting a major block in endocytosis (Fig. 1A). Trypanosomes were exposed to different UDA concentrations for 10 h or 24 h in order to monitor the effect on morphology and growth. Morphological defects and lysis were quickly observed coinciding with lectin binding (20 min). However, after 10 h of treatment, a high percentage of lysis was found at concentrations of 1.125 μM and higher and, after drug removal, growth was resumed at all tested concentrations. In contrast, exposure to UDA for 24 h induced practically 100% of cell lysis at UDA concentrations of 0.565 μM and higher, and the effect was irreversible at concentrations higher than 1.125 μM (Fig. 1B). In summary, UDA acts as a trypanocidal agent when parasites are exposed to 5-fold the EC_50_ for 24 h.

A more detailed study of the morphology and the distribution of nuclei and kinetoplasts was performed in cells exposed to different concentrations of UDA for 10 h by DAPI staining. At a dose equivalent to 5-fold the EC_50_ (1.125 μM), the microscopic analysis revealed a substantial accumulation of trypanosomes which have completed mitosis (2N2K) (53%) and the emergence of a significant number of cells with multiple nuclei and kinetoplasts (XNXK) (17%), both indicative of a severe defect in cytokinesis which would lead to multinucleated cells after successive rounds of replication (Fig. 1C and D). Additionally, significant alterations in morphology were detected in the population of cells treated with UDA consisting in the presence of enlarged and rounded cells with detachment or loss of the flagellum (50% of the total cells at 1.125 μM) (Fig. 1D).

### UDA selection pressure induces changes in the parasite surface glycoproteins as a mechanism of resistance

To analyze further the mode of action of UDA, we generated several resistant cell lines by stepwise selection to escalating concentrations of UDA. Two different lines of resistant cells designated UDA15a and UDA15b were obtained at the end of the adaptation process to the highest concentration, showing resistance selectivity indices of 9.2 and 4.8, respectively (Table 1). When resistant strains were grown in the absence of drug for three months, their resistance indices remained unaltered (Table 1), therefore indicating that the resistance phenotype is stable and genetically encoded.

**Table 1:** EC_50_ values for UDA in parental (BSF) and UDA-resistant *T*. *brucei* bloodstream lines.

	Cell line	EC_50_ (μM)	R-index^a^
	Tb BSF	0.225 ± 0.006	1.0
**UDAa**	UDA3.5a	0.46 ± 0.02	2.0
	UDA5a	0.84 ± 0.01	3.7
	UDA10a	1.24 ± 0.02	5.5
	UDA10a p-Rem (3M)^b^	1.53 ± 0.04	6.8
	UDA15a	2.08± 0.03	9.2
	UDA15a p-Rem (3M)^b^	2.26 ± 0.03	10.0
**UDAb**	UDA3.5b	0.39 ± 0.02	1.7
	UDA5b	0.66 ± 0.02	2.9
	UDA10b	0.90 ± 0.01	4.0
	UDA10b p-Rem (3M)^b^	1.05 ± 0.02	4.7
	UDA15b	1.09 ± 0.02	4.8
	UDA15b p-Rem (3M)^b^	1.345 ± 0.001	6.0

Resistance indices (R-index) for UDA-resistant cells with respect to the parental line are indicated.

^a^Resistance selectivity index or the ratio of EC_50_ UDA-resistant cells/EC_50_ parental cells.

^b^Post-removal time period of cell cultivation in the absence of UDA is indicated between parentheses.

**Fig 2:**
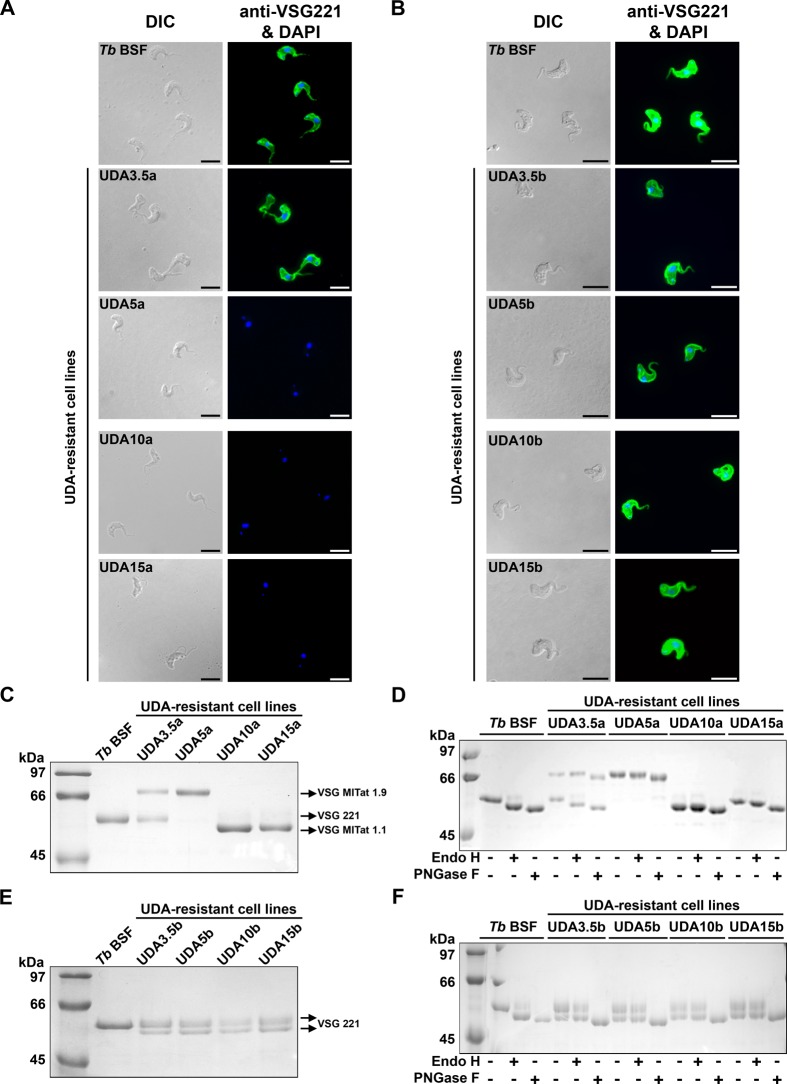
Analysis of the VSG expressed in UDAa and UDAb resistant lines. A and B) VSG221 expression in parental and UDA-resistant parasites was monitored by immunofluorescence microscopy using a polyclonal antibody against VSG221. Nuclear and kinetoplast DNA was stained with DAPI. Bars, 10 μm. C and E) sVSG were purified from parental and resistant lines as described [29,30] and analyzed by SDS/PAGE and Coomassie blue staining. D and F) sVSG samples were digested with Endo H (that removes oligomannose *N*-linked glycans) or PNGase F (that removes all *N*-linked glycans), and the products of the reaction were subjected to SDS/PAGE and Coomassie blue staining.

Given that parasite surface glycoproteins are the possible target for UDA, we next performed a series of experiments aimed at establishing the identity and glycosylation status of the VSGs in different resistant strains. Indirect immunofluorescence analysis showed that UDA pressure resulted in at least one VSG switching event during the selective process between the UDA3.5a and UDA5a cell lines (Fig. 2A). The sVSGs expressed in the different strains were purified, subjected to SDS/PAGE, stained with Coomassie Blue, and identified by tryptic peptide mass fingerprinting using MALDI-TOF analysis (Voyager DE PRO, AB Sciex). As previously reported, the parental cell line expresses a single VSG (VSG221) which is resolved as a single band of ≈54 kDa that is *N*-glycosylated at Asn263 with paucimannose structures (Endo H-resistant site) and at Asn428 with oligomannose structures (Endo H-sensitive site) [6]. In the UDA3.5a resistant strain, sVSG appears as two bands corresponding to two different VSGs identified as VSG221 (lower band) and VSG MITat 1.9 (VSG VO2) (upper band), the latter also expressed in the UDA5a strain. On the other hand the UDA10a and UDA15a strains expressed a predominant VSG of approximately 50 kDa and a less abundant band of higher molecular mass, both identified as VSG MITat 1.1 (VSG 060) (Fig. 2C) indicating additional VSG selection events. On the contrary, all UDAb-resistant strains were positively probed with anti-TbVSG221 (Fig. 2B). However, the analysis by SDS-PAGE and MALDI-TOF revealed the existence of two VSG221 forms probably due to changes in the *N*-glycosylation profile (Fig. 2E). We suggest that two glycoforms of VSG221 coexist: the upper band would correspond to the form with two *N*-glycosylation sites occupied, whereas the lower band would only have one of the sites modified by glycosylation (Asn263). A similar glycosylation status was reported in a previous study of parasites resistant to the peptidic CBA HHA [19]. In order to gain insight into the glycosylation pattern of the VSGs expressed in both resistant lines, an endoglycosidase digestion was carried out with Endo H that removes only conventional triantennary oligomannose and hybrid *N*-glycans or PNGase F that removes all types of *N*-glycans. The VSG221 expressed in UDA3.5a harbors Endo H-resistant and Endo H-sensitive *N*-glycosylation sites, in a similar manner to the VSG221 of the parental line. The VSG MITat 1.9 expressed in UDA3.5a and UDA5a strains has four asparagines susceptible for *N*-glycosylation, but only one of them appears to be glycosylated with structures resistant to Endo H. The VSG MITat 1.1 expressed in UDA10a and UDA15a has two probable *N*-glycosylation sites. Treatment with Endo H produced a molecular mass shift only in the VSG of UDA15a, while PNGase F appears to release one or two *N*-glycans from UDA10a and UDA15a VSGs, respectively. Thus, UDA10a VSG has only one Endo H-resistant *N*-glycan while the sVSG purified from UDA15a has Endo H-resistant and Endo H-sensitive *N*-glycans (Fig. 2D).

As mentioned above, UDAb-resistant cell lines expressed two VSG221 glycoforms. In this case, the *N*-glycans at both glycosylation sites were Endo H-resistant (Fig. 2F), which indicates a change from oligomannose to paucimannose structures. We investigated whether the absence of Endo-H-sensitive glycans could be caused by the lack of any of the asparagines. However, a sequencing analysis showed that all asparagines susceptible of being *N*-glycosylated were present in VSG221 as well as in VSG MITat 1.1 from resistant strains, thus ruling out mutations affecting the asparagines as responsible for hypoglycosylation.

**Fig 3:**
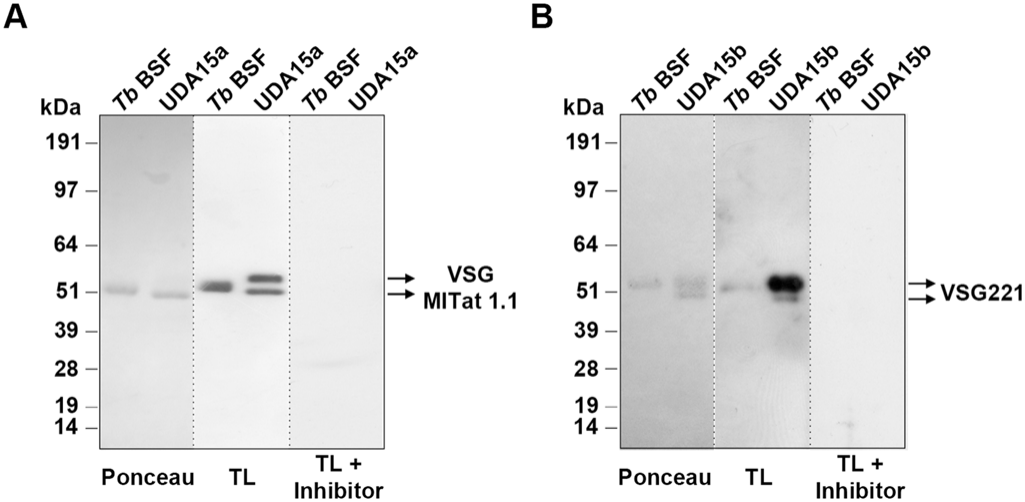
Assessment of the VSG glycosylation status by lectin blotting. Purified sVSG of UDA15a (A) and UDA15b (B) were probed with tomato lectin (TL). Ponceau staining was used as loading control and chitin hydrolysate (inhibitor) was used as a specificity control for TL.

To assess the nature of the *N*-glycans in the sVSG, we performed a lectin blotting using tomato lectin (TL) that recognizes mainly poly-*N*-acetyl lactosamines [32]. TL has also been shown to bind to the residual Man_3_GlcNAc_2_ [Manα1–3(Manα1–6)Manβ1–4GlcNAcβ1–4GlcNAc] pentasaccharide of complex *N*-glycans treated with sialidase, β-galactosidase, and β-hexosaminidase and to the exposed ManGlcNAc_2_ (Manβ1–4GlcNAcβ1–4GlcNAc) trisaccharide core of oligomannose *N*-glycans treated with α-mannosidase [33]. However, a fully trimmed triantennary Man_5_GlcNAc_2_ is not a TL ligand [34]. A TL inhibitor, chitin hydrolysate, was included in the study as specificity control. The resulting blotting corroborated the existence in UDA15a of two glycoforms of VSG MITat 1.1 and the labeling intensity suggested an increase in poly-*N*-acetyl lactosamine, or abbreviated core structures derived from paucimannose *N*-glycans (Fig. 3A). With regard to the VSG221 glycoforms expressed in UDA15b, the intensity of the upper band after TL blotting suggests again an enrichment of Endo H-resistant *N*-glycans containing large *N*-acetyl lactosamine repeats or paucimannose derived structures (Fig. 3B). In summary, our results show that *T*. *brucei* is able to overcome UDA selective pressure by different means in order to reduce the lectin-binding. In the present study both VSG switching and modifications of the VSG *N*-glycosylation profile take place.

### UDA-resistant parasites exhibit defects in oligosaccharyltransferases

**Fig 4:**
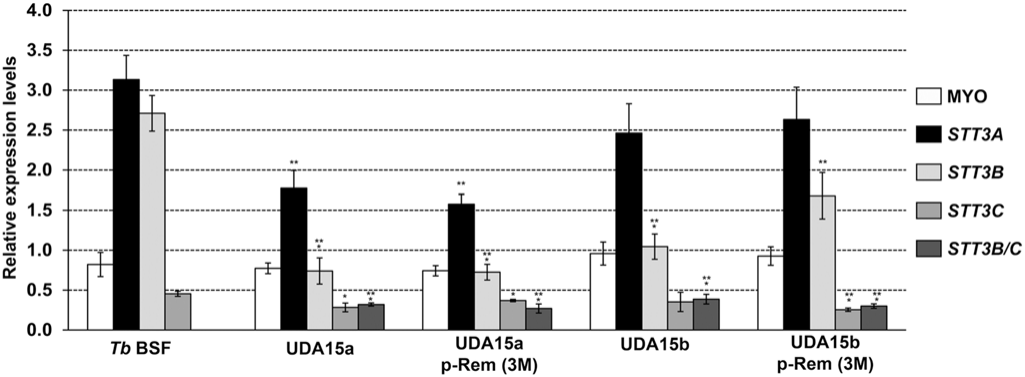
Relative expression of *TbSTT3* genes in UDAa and UDAb-resistant cell lines. mRNA were purified from parental and resistant lines in the presence or the absence of UDA selective pressure (p-Rem 3M), quantified by RT-qPCR and normalized to the expression of *PI3K-like* gene. *Myosin 1B* gene expression was also introduced in the analysis as control. Values were calculated from triplicates of three independent experiments. The asterisks show significant differences by the Student’s *t*-test (n = 3). *, *p* < 0.05, **, *p* < 0.01 and ***, *p* < 0.001 vs the parental strain.

In a previous work, we demonstrated that resistance to the lectin HHA was achieved through genetic rearrangements that affected the expression of the genes that code for OSTs [19]. To examine the implication of the same activities in the resistance to UDA, mRNA expression levels of the *TbSTT3A*, *TbSTT3B* and *TbSTT3C* genes as well as the expression of potential chimeric genes resulting from genetic recombination, were evaluated by RT-qPCR. Primers used in the analysis were designed against the variable region of each gene (S1A Fig) [19]. Relative expression of mRNA levels was calculated relative to the expression level of the *PI3K-like* gene. In UDA15a cells, *TbSTT3A*, *TbSTT3B* and *TbSTT3C* mRNA expression levels were reduced 1.8, 3.7 and 1.6-fold, respectively. In the resistant line UDA15b, the expression of *TbSTT3B* was diminished 2.7-fold, while *TbSTT3A* and *TbSTT3C* mRNA levels remained unaltered. In addition, resistant lines also expressed low levels of a chimeric gene comprised by *TbSTT3B* at the 5’-end and *TbSTT3C* at the 3’-end (*TbSTT3B/C*) (Fig. 4). The existence of the *TbSTT3B/C* fusion gene was further confirmed by PCR using genomic DNA from resistant cells as template (S1B Fig).

In addition, the nucleotide sequence of canonical *TbSTT3* genes (A, B and C) and their corresponding 5’UTRs and 3’UTRs were determined in order to verify their integrity and are shown in S1 Table. TbSTT3A from the UDA15a strain showed two amino acid changes: K513E and T725A. In TbSTT3B, both UDA15a and UDA15b strains express a mutated protein with the changes N627S and E629K. An additional amino acid change (T633N) was present in UDA15b. Regarding TbSTT3C, no changes were detected in any of the strains. S2 Table summarizes the mutations found in the UTRs regions. Further analysis is currently underway to assess the functional significance of the mutations affecting the *TbSTT3* genes.

### UDA-resistant parasites exhibit reduced infectivity and virulence in a mouse model

**Fig 5:**
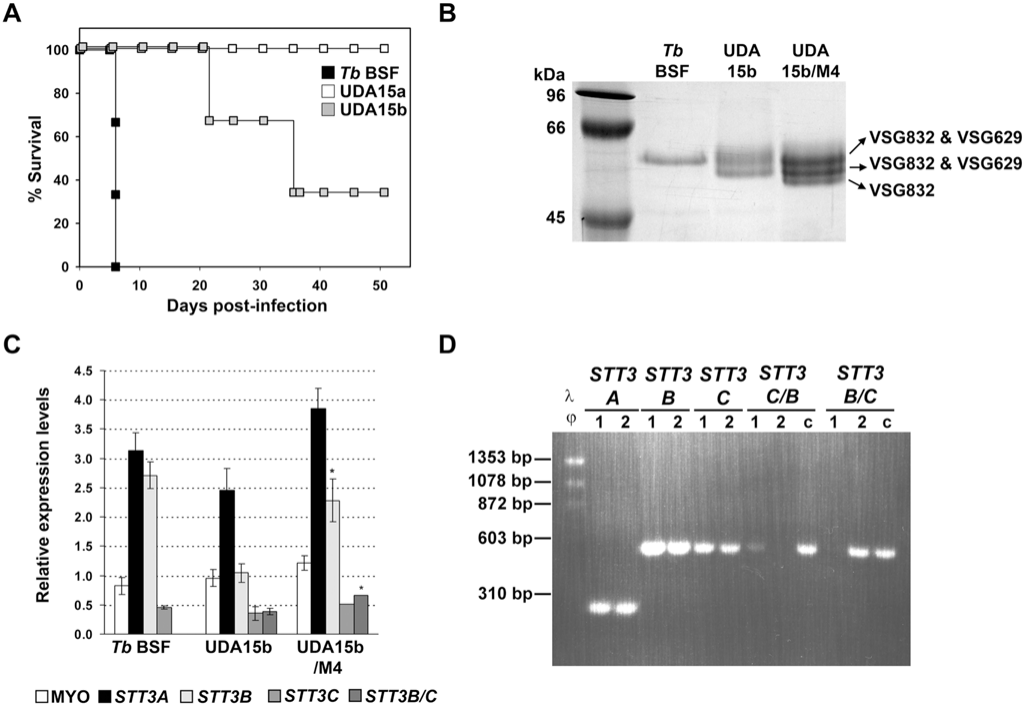
Survival of mice inoculated with UDA-resistant parasites. A) Kaplan-Meier survival analysis of mice infected with parental (*Tb* BSF), and UDA15a and UDA15b resistant parasites. B) SDS/PAGE analysis of sVSG expressed in UDA15b parasites isolated from mouse #4 (UDA15b/M4) at the final point of infection. Coomassie blue stained bands were identified by MALDI-TOF analysis. C) Relative expression of *TbSTT3* genes in UDA15b/M4 was determined by RT-qPCR. The asterisks indicate significant differences according to the Student’s *t*-test (n = 3). *, *p* < 0.005 vs the UDA15b strain. D) PCR analysis of full-length and chimeric *TbSTT3* genes in genomic DNA from (1) *Tb* BSF, (2) UDA15b/M4 and (c) HHA20 as control [19].

To evaluate the effect of altered OST expression on VSG glycosylation and the subsequent capacity of trypanosomes to survive and grow in the mammalian host, Balb/C mice were inoculated with parental, UDA15a or UDA15b strains and the course of the infection was followed by monitoring parasitaemia. While mice infected with the parental line died at 6 days post-infection when parasitaemia reached 10^9^ parasites/mL, UDA15a trypanosomes were not able to infect and kill mice during the monitored period (2 months) (Fig. 5A). In the case of mice inoculated with UDA15b resistant trypanosomes, the onset of parasitaemia was delayed from day 3 (mice infected with control trypanosomes) until day 14 post-infection. These mice presented cycles of parasite clearance followed by severe relapses of parasitaemia (≈10^7^ parasites/mL) that eventually led to animal death.

UDA15b parasites were harvested at the final stage of the infection from one of the mice to examine the identity of the expressed VSG, determine their *TbSTT3* mRNA levels and reevaluate the resistance to UDA. The isolated parasites, renamed UDA15b/M4 (animal dead at 21 days post-infection), expressed a novel set of VSGs that appeared as three discrete bands on SDS-PAGE. MALDI-TOF analysis identified bands 1 and 2 as a mixture of VSG832 and VSG629, while band 3 consisted exclusively of VSG832 (Fig. 5B). The RT-PCR analysis showed that *TbSTT3B* mRNA levels were fully restored in these parasites, which also presented an increase in the expression of the chimeric *TbSTT3B/C* (Fig. 5C). The presence of chimeric *TbSTT3B/C* and canonical *TbSTT3* genes in the genome was analyzed by PCR (Fig. 5D). Despite the changes described above, mouse blood-recovered trypanosomes UDA15b/M4 preserved the UDA-resistant phenotype (R-index = 3.7) at a similar level to that of UDA15b parasites (R-index = 4.8).

### General defects in protein glycosylation are associated with UDA-resistance phenotypes

Since one of the mechanisms involved in the resistance to lectins is the altered expression of OST activities, we expected significant changes in the whole spectrum of glycoproteins, not only VSGs. To assess whether the defective glycosylation was extended to other glycoproteins, we performed a lectin blotting against whole cell lysates of UDA15a and UDA15b with TL and ConA that binds oligomannose and hybrid glycans containing Manα1–3(Manα1–6)Manα1 [35] as probes. Chitin hydrolysate and α-methyl mannopyranoside inhibitors of TL and ConA, respectively, were included in the study as specificity controls.

**Fig 6:**
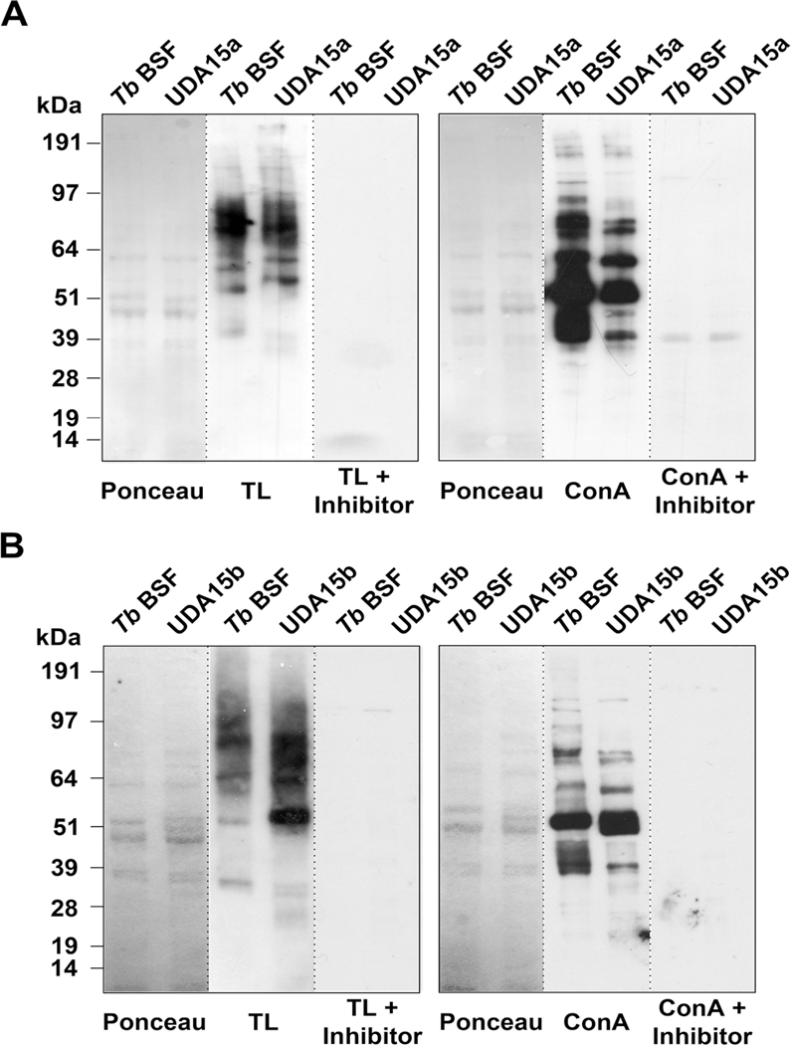
Changes in the glycosylation profile of UDA-resistant cell lines. Whole cell extracts obtained from UDA15a (A) and UDA15b (B) after hypotonic lysis were probed with TL or ConA. Ponceau staining was used as loading control. Chitin hydrolysate (inhibitor) and α-methylmannopyranoside were used as a specificity control for TL or ConA, respectively.

In UDA15a parasites, the repertoire of glycoproteins detected by TL was different to that of the parental line although the overall signal intensity was similar in both lines (Fig. 6A, left panel). In contrast, the blotting with ConA showed a significant reduction in the proportion of glycoproteins present in the extract which are detected by this lectin (Fig. 6A, right panel). The UDA15b strain contains a higher amount of TL-specific glycoproteins than parental cells, while slight differences in the identity and number of ConA-binding glycoproteins were observed (Fig. 6B). Taken together, these results indicate that while UDA15a parasites are mainly dealing with glycosylation defects that reduce the *N*-glycans of oligomannose-type structures, in UDA15b parasites, such defects result in an increase in complex paucimannose-derived *N*-glycans.

### Lectin-binding and endocytic capacity is impaired in resistant cell lines

**Fig 7:**
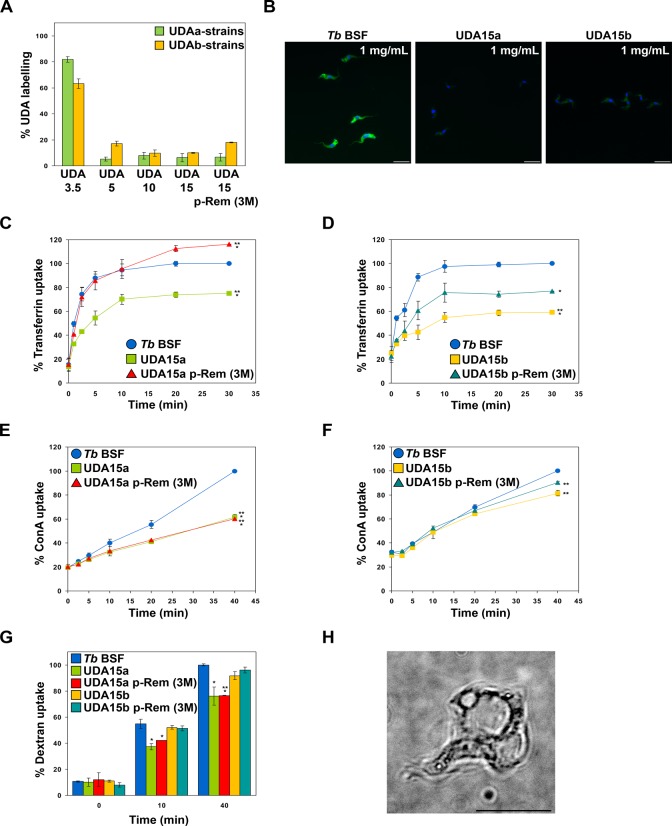
Resistant parasites exhibit deficient binding and uptake of UDA-FITC. A) Plot showing binding of UDA to resistant lines grown in the presence or after 3 months of lectin removal (p-Rem 3M). Data were represented as the percentage of binding relative to the labeling of the parental line. B) Microscopy images of fixed cells showing the decrease in UDA-labeling of UDA15a and UDA15b cells in comparison to parental *T*. *brucei* BSF. Nuclear and kinetoplast DNA were stained with DAPI. Bars, 10 μm. C and D) Plots showing the uptake of transferrin in UDA15a and UDA15b lines, respectively. E and F) Uptake of ConA in UDA15a and UDA15b lines, respectively. G) Uptake of dextran. H) Live cell microscopy illustrates the “big-eye” phenotype resulting from an endocytosis block after treatment of parental *T*. *brucei* BSF with 1.125 μM UDA for 10 hours. Uptake percentages were calculated relative to the maximum uptake observed in the parental line (*Tb* BSF). The asterisks show significant differences by the Student’s *t*-test (n = 3). *, *p* < 0.05, **, *p* < 0.01 and ***, *p* < 0.001 vs the parental strain.

A detailed study on lectin binding and endocytosis was performed to provide detail on the mechanism of UDA killing and parasite adaptation to UDA. Parasites were labeled with UDA-FITC, transferrin-FITC, ConA-AlexaFluor 594 conjugate or Alexa Fluor 488-labeled dextran. After exhaustive washing, the fluorescence signal was measured by FACS or analyzed by 3D microscopy. In the first place, parental and UDA-resistant strains were labeled with UDA-FITC for 30 min as described in Materials and Methods. Notably, all UDA-resistant strains, with the exception of UDA3.5a and UDA3.5b, had lost most of their capacity to bind the lectin, which remained between 5% and 20% with regard to the UDA-binding capacity of the parental line (Fig. 7A). Additionally, a fluorescence microscopy analysis was carried out on fixed UDA15a and UDA15b resistant cells. Microscopy images showed a profuse UDA-labeling of the parasite surface in the parental line while in UDA15a and UDA15b, the fluorescence signal was almost undetectable (Fig. 7B). These results further support that UDA indeed binds VSG221 and that the resistance phenotype is related to a reduced lectin-binding to surface glycoproteins as a result of VSG switching and/or changes in the expression of oligosaccharyltransferases, thus giving rise to variants with different glycans.

The endocytic capacity of UDA-resistant mutants was evaluated by using transferrin as receptor-mediated endocytosis marker, mannose-binding lectin ConA as a marker for membrane-bound endocytic activity [36] and dextran to measure fluid-phase endocytosis [37]. Transferrin uptake was significantly reduced in both UDA15a and UDA15b resistant lines. However, when parasites were grown in the absence of UDA selective pressure for 3 months, endocytosis was completely or partially restored in UDA15a or UDA15b cells, respectively (Fig. 7C and D). ConA uptake was strongly impaired in UDA15a with respect to the parental line, although it might be due to the presence of a different surface glycoprotein (VSG MITat 1.1) with lower capacity than VSG221 to bind ConA in these parasites (Fig. 7E). In the case of UDA15b, we only observed a slight defect in ConA internalization (Fig. 7F). Finally, dextran uptake was normal in UDA15b but significantly and irreversibly decreased in UDA15a (Fig. 7G). In the parental line endocytosis defects after UDA treatment were further confirmed by live microscopy analysis which revealed cells exhibiting the characteristic “big-eye” phenotype produced by an enlargement of the flagellar pocket and represented 13.6% of the total population when exposed to 5-fold (1.125 μM) the EC_50_ for 6 h (Fig. 7H). We propose that the mechanism of cell death of UDA is through membrane rupture due to the pressure on the cell as the cytoplasm becomes compressed by the enlarging pocket as previously described for cells undergoing endocytosis defects [38].

### VSG switching occurs under UDA selective pressure

As reported above, at least two changes in the expression of the predominant VSG took place during the generation of one of the UDA-resistant parasite lines. First, the parental line VSG221 switched to the telomeric VSG MITat 1.9 (Tb427.BES129.14), and subsequently, to the subtelomeric VSG MITat 1.1. To understand the nature of this observation, a variable region located downstream of the active VSG promoter was amplified by PCR using cDNA from resistant and parental lines. Sequencing of the amplified region showed no differences between parental and resistant lines (S2 Fig), thus concluding that expression of a different VSG involved the insertion of a silent VSG gene into the active expression site of VSG221.

### Resistance to UDA involves cross-resistance to other carbohydrate binding agents

In order to determine whether UDA-resistant mutants exhibited cross-resistance to other CBAs with different substrate specificities, UDA15a and UDA15b trypanosomes were cultured with increasing concentrations of HHA, EHA, GNA and NPA and the EC_50_ values for each compound were determined (Table 2). Cross-resistance indices (R-index) were calculated as the ratio of EC_50_ in UDA15 cells/EC_50_ in parental cells. The UDA15a strain exhibits an overall cross-resistance to other CBAs with R-index values >20. In the case of UDA15b, a very high cross-resistance was observed specifically for the mannose-specific lectin EHA (74.6), whereas the remaining CBAs produced R-index values between 9.7 and 26.9. Indeed, the high cross-resistance to EHA further supports the conclusion that glycoproteins in resistant cell lines are enriched in paucimannose-derived *N*-glycans in disadvantage of oligomannose residues.

**Table 2:** EC_50_ values and cross-resistance (R) indices of UDA15a and UDA15b resistant lines.

CBAs	Specificity	EC_50_ *T*. *b* BSF (μM)	EC_50_ UDA15a (μM)	R-index^a^	EC_50_ UDA15b (μM)	R-index^a^
HHA	α(1,3)- α(1,6) Man	0.020 ± 0.001	0.50 ± 0.02	25.0	0.254 ± 0.004	12.7
EHA	Man	0.0142 ± 0.0003	0.55 ± 0.03	38.7	1.06 ± 0.03	74.6
GNA	α(1,3) Man	0.043 ± 0.002	1.6 ± 0.1	37.2	1.16 ± 0.03	26.9
NPA	α(1,6) Man	0.058 ± 0.001	1.2 ± 0.2	20.7	0.558 ± 0.006	9.7

## Discussion

The present study aimed to examine the properties of carbohydrate-binding agents as trypanocidal agents, their mode of action and the potential pathways that these organisms may employ to generate resistance. In contrast with previous studies showing that a variety of lectins do not affect viability of bloodstream forms of *T*. *brucei* [31], here we report that the GlcNAc-specific lectin UDA (*Urtica dioica* agglutinin), one of the smallest monomeric plant lectins, binds irreversibly to the surface coat of *T*. *brucei* with a cytotoxic activity at concentrations close to the EC_50_ (0.225 μM). As an antiviral agent, nanomolar concentrations of UDA have been shown to strongly bind to HIV-1 gp120, thus inhibiting virus infection by blocking virus entry and viral transmission upon co-cultivation of persistently HIV-infected and uninfected cells [23,39,40]. The present evidence strongly suggests that UDA binds to surface glycoproteins predominantly VSG221. Although the UDA-VSG221 dissociation constant was not determined in this work, we hypothesize that the lectin exhibits a high affinity and binds tightly to the surface coat glycoproteins and is deficiently internalized, accumulating with time in the flagellar pocket. We propose that UDA’s trypanocidal activity is favored by its high affinity for the glycoprotein and also by its low molecular weight, which grants the lectin full accessibility to its physiological target. Both attributes are shared by VSG-specific nanobodies (Nsbs) and have been reported to play an essential role in its cytotoxic action [41]. Furthermore, it is probable that any moiety that irreversibly binds to the VSG coat would impair endocytosis and result in cytotoxicity.

Trypanosome exposure to UDA induces profound defects in endocytosis, cytokinesis stalling, morphological cell abnormalities characterized by the appearance of big and rounded cells, accumulation of the lectin and enlargement of the flagellar pocket and of cell vesicles; a phenotype largely reminiscent of that induced by treatment with the (α1,3)/(α1,6)-mannose-specific *Hippeastrum* hybrid agglutinin (HHA) [19]. A stalled cytokinesis has been previously observed in cells where VSG was depleted by RNAi, together with differences in cell morphology and also in the relative position of kinetoplast and nucleus [42]. In the present study the population of 2N2K cells showed mostly a rounded morphology, in certain cases with a loss of the flagellum, together with trypanosomes exhibiting two parallel flagella. Similar defects in the flagellar pocket were also found after RNAi-mediated down-regulation of clathrin heavy chain or phosphatidylinositol 4-kinase β in procyclic forms and are an indication of defects in the endocytic pathway [38,43]. We hypothesize that UDA binds to VSGs and other glycoproteins located in the flagellar pocket severely blocking endocytosis. Subsequently, glycoprotein-UDA complexes are deficiently internalized with only a minor fraction of them being endocytosed. As a consequence, these complexes tend to accumulate in the flagellar pocket with time, further blocking the endocytic process and causing a build-up of the internal pressure that might eventually lead to cell death.

In order to gain insight into the molecular basis of UDA’s mode of action we generated resistant parasite strains. The resistance phenotype was characterized by a reduced capacity of VSGs to bind UDA either due to defective glycosylation or to VSG switching events that led to the predominant expression of novel VSGs with low affinity for the lectin. Because this phenotype was stable and remained in the absence of UDA’s selective pressure, we concluded that the resistance phenotype is genetically encoded. Evidence obtained from different approaches including glycosidase digestion, lectin blotting and cross-resistance assays suggests that resistance to UDA is specifically conferred by the presence of VSGs which are primarily *N*-glycosylated with paucimannose-type structures at the expense of oligomannose structures. Similar alterations in glycosylation have been previously reported for *TbGlcaseIIα* null mutants [5], *TbUAP* conditional null mutant cells [44], *TbALG3* null mutants [6], TbSTT3B knockdowns [7], GDP-Man PP RNAi cells [45] and HHA-resistant mutants [19]. *T*. *brucei* glycosylates VSGs in a site-specific manner by the action of two catalytic OSTs. The STT3A activity transfers the Man_5_GlcNAc_2_-PP-Dol to asparagines flanked by an acidic sequence yielding paucimannose structures, whereas STT3B catalyzes the transfer of Man_9_GlcNAc_2_-PP-Dol to any remaining asparagine giving rise to oligomannose *N*-glycans [5,6]. Therefore, a strong down-regulation of *TbSTT3B* and/or a switch to a different VSG with novel glycosylation sites are the genetic factors involved in the emergence of parasites with surface glycoproteins rich in paucimannose *N*-glycans instead of oligomannose structures. It is worth emphasizing at this point that regardless the type of structure, the reason behind the drastic reduction in affinity is likely the nature or the accessibility of the novel glycan residues covalently attached to the VSGs. Importantly, whereas *N*-glycosylation defects did not affect proliferation *in vitro*, resistant parasites either were not infective or virulence was significantly attenuated in a murine model of infection. The vital role of VSG-glycosylation in host-parasite interactions has been demonstrated in previous work where RNAi-mediated or HHA-induced TbSTT3B knock-downs exhibited reduced virulence [7,19].

The differences in resistance modalities observed for UDA when compared with HHA [19] can be due to differences in sugar specificity of both lectins. Whereas HHA is mainly targeting α(1,3)-α(1,6)-mannose oligomers, UDA strongly interacts with (GlcNAc)_3_-oligomers in addition to high mannose *N*-glycans such as Man9, Man8, Man6 and Man 5 [46]. Such different specificities may therefore eventually determine different interaction sites of UDA and HHA on parasite glycans.

It should also be emphasized that lectins in general may also bind to mammalian glycoproteins and can display pronounced mitogenic effects. However, the binding specificities and potential toxic side-effects may differ considerably between different lectins. For example, whereas the α(1,2)-mannose-specific lectin cyanovirin N is highly mitogenic and induces a wide variety of chemokines, microvirin, targeting α(1,2)-mannose oligomers as well, was shown to have a much higher safety profile [47]. It should also be recognized that the clustered occurrence of glycans on parasite VSG and the presence of paucimannose glycans clearly differ from the glycans in glycoproteins of mammalian cell membranes, indicating a higher selectivity than anticipated of some lectins to discriminate between parasitic and mammalian cells. Such discrimination of CBAs between host and parasite has also been observed for CBA interaction with the human immunodeficiency virus [18]. Of course, the occurrence of potential side-effects should be monitored and investigated in animal models prior to any clinical application of a particular CBA.

In the present study, we have conducted a detailed investigation of the cytotoxic activity and mode of action of *Urtica dioica* agglutinin on the bloodstream form of *T*. *brucei*. We present evidence indicating that the lectin binds mainly to surface VSGs, although other glycoproteins cannot be discarded. However, the exact molecular mechanism responsible for the resulting cell cycle defects and switching events remains to be established. Prolonged exposure to UDA can generate resistant mutants through different strategies whose ultimate goal is to alter the glycosylation status of the parasite surface and thus, reduce lectin-binding. However, a major drawback of the adaptation to UDA for the parasite is the loss of infectivity and virulence. Taken together, these observations suggest that carbohydrate binding agents that interact with VSG glycans could be explored as a novel avenue to design antitrypanosomal agents.

## Supporting Information

S1 Fig STT3 genes rearrangement. A) Scheme of *TbSTT3* genes and primers used in the study. B) PCR analysis of the full-length and chimeric *TbSTT3* genes. Target sequences were amplified from UDAa and UDAb genomic DNAs with different primer combinations according to the scheme shown in panel A.
(TIF)

S2 Fig Alignment of the active VSG promoter sequence from the UDAa-resistant strains. The alignment was performed with ClustalW2 (EMBL-EBI).
(TIF)

S1 Table Oligosaccharyltransferases amino acid changes encoded by *TbSTT3A*, *TbSTT3B* and *TbSTT3C* genes in UDA-resistant strains. Changes were referred to the amino acid sequences of the parental line obtained in our laboratory.
(DOCX)

S2 Table Mutations found in the UTRs of the *TbSTT3A*, *TbSTT3B* and *TbSTT3C* genes in UDA-resistant strains. Changes were referred to the sequences of the parental line obtained in our laboratory.
(DOCX)
