# Supplementary material for: Exposure of Trypanosoma brucei to an N-acetylglucosamine-Binding Lectin Induces VSG Switching and Glycosylation Defects Resulting in Reduced Infectivity
Source: PLoS Negl Trop Dis. 2015 Mar 6;9(3):e0003612. doi: 10.1371/journal.pntd.0003612 (PMC4351956; doi:10.1371/journal.pntd.0003612)
Supplement: S1 Table — Changes were referred to the amino acid sequences of the parental line obtained in our laboratory. (DOCX) [file pntd.0003612.s003.docx]

**Table S1. Oligosaccharyltransferases amino acid changes encoded by *TbSTT3A*, *TbSTT3B* and *TbSTT3C* genes in UDA-resistant strains.**

Changes were referred to the amino acid sequences of the parental line obtained in our laboratory.

|  | ***TbSTT3A*** | ***TbSTT3B*** | ***TbSTT3C*** |
| --- | --- | --- | --- |
| **UDA 15a** | K513E | N627S | No changes |
|  | T725A | E629K |  |
| **UDA15b** | No changes | N627S | No changes |
|  |  | E629K |  |
|  |  | T633N |  |
