# Supplementary material for: Exposure of Trypanosoma brucei to an N-acetylglucosamine-Binding Lectin Induces VSG Switching and Glycosylation Defects Resulting in Reduced Infectivity
Source: PLoS Negl Trop Dis. 2015 Mar 6;9(3):e0003612. doi: 10.1371/journal.pntd.0003612 (PMC4351956; doi:10.1371/journal.pntd.0003612)
Supplement: S2 Table — Changes were referred to the sequences of the parental line obtained in our laboratory. (DOCX) [file pntd.0003612.s004.docx]

**Table S2. Mutations found in the UTRs of the *TbSTT3A*, *TbSTT3B* and *TbSTT3C* genes in UDA-resistant strains.**

Changes were referred to the sequences of the parental line obtained in our laboratory.

|  | ***STT3A 5’UTR*** | ***STT3A 3’UTR or***  ***STT3B 5’UTR*** | ***STT3B 3’UTR or***  ***STT3C 5’UTR*** | ***STT3C 3’UTR*** |
| --- | --- | --- | --- | --- |
| **UDA 15a** | No mutations | No mutations | No mutations | T949A |
| **UDA15b** | No mutations | No mutations | C435T | G743A |
|  |  |  |  | C744G |
